# Molecular Typing of *Staphylococcus aureus* Isolated from Patients with Autosomal Dominant Hyper IgE Syndrome

**DOI:** 10.3390/pathogens6020023

**Published:** 2017-06-06

**Authors:** Inka Sastalla, Kelli W. Williams, Erik D. Anderson, Ian A. Myles, Jensen D. Reckhow, Marlene Espinoza-Moraga, Alexandra F. Freeman, Sandip K. Datta

**Affiliations:** 1 Bacterial Pathogenesis Unit, Laboratory of Clinical Infectious Diseases, National Institute of Allergy and Infectious Diseases, National Institutes of Health, Bethesda, MD 20892, USA; sastallai@niaid.nih.gov (I.S.); kelli.williams@nih.gov (K.W.W.); erik.anderson2@nih.gov (E.D.A.); mylesi@niaid.nih.gov (I.A.M.); jensen.reckhow@gmail.com (J.D.R.); 2 Tuberculosis Research Section, Laboratory of Clinical Infectious Diseases, National Institute of Allergy and Infectious Diseases, National Institutes of Health, Bethesda, MD 20892, USA; marlene.espinozamoraga@nih.gov; 3 Immunopathogenesis Section, Laboratory of Clinical Infectious Diseases, National Institute of Allergy and Infectious Diseases, National Institutes of Health, Bethesda, MD 20892, USA; freemaal@mail.nih.gov

**Keywords:** *Staphylococcus aureus*, Job’s Syndrome, STAT3, multi-locus sequence typing

## Abstract

Autosomal dominant hyper IgE syndrome (AD-HIES) is a primary immunodeficiency caused by a loss-of-function mutation in the Signal Transducer and Activator of Transcription 3 (STAT3). This immune disorder is clinically characterized by increased susceptibility to cutaneous and sinopulmonary infections, in particular with *Candida* and *Staphylococcus aureus*. It has recently been recognized that the skin microbiome of patients with AD-HIES is altered with an overrepresentation of certain Gram-negative bacteria and Gram-positive staphylococci. However, these alterations have not been characterized at the species- and strain-level. Since *S. aureus* infections are influenced by strain-specific expression of virulence factors, information on colonizing strain characteristics may provide insights into host-pathogen interactions and help guide management strategies for treatment and prophylaxis. The aim of this study was to determine whether the immunodeficiency of AD-HIES selects for unique strains of colonizing *S. aureus*. Using multi-locus sequence typing (MLST), protein A (spa) typing, and PCR-based detection of toxin genes, we performed a detailed analysis of the *S. aureus* isolates (*n* = 13) found on the skin of twenty-one patients with AD-HIES. We found a low diversity of sequence types, and an abundance of strains that expressed methicillin resistance, Panton-Valentine leukocidin (PVL), and staphylococcal enterotoxins K and Q (SEK, SEQ). Our results indicate that patients with AD-HIES may often carry antibiotic-resistant strains that harbor key virulence factors.

## 1. Introduction

*Staphylococcus aureus* colonizes human mucosa and skin and is a major human pathogen that causes a strikingly broad range of infections, ranging from relatively minor skin abscesses to potentially life-threatening pneumonia, osteomyelitis, endocarditis, necrotizing fasciitis, septicemia, and toxic shock syndrome [1]. The multitude of virulence factors and immune response modulators expressed by *S. aureus* contribute to these diverse disease manifestations [2]. In addition, rapid adaptation and development of bacterial resistance to antibiotics impede the effective treatment of *S. aureus* infections, as demonstrated by the epidemic emergence of community-associated methicillin-resistant *S. aureus* (CA-MRSA) strains outside the hospital setting [3,4]. The association of certain clonal groups of MRSA, such as USA300 [5], with outbreaks of infection suggests a critical impact of strain-dependent virulence factor expression.

Although *S. aureus* can infect otherwise healthy individuals, the increased susceptibility of patients with certain primary immunodeficiencies provides insight into the components of a protective immune response. For example, the critical role of neutrophils is highlighted by the increased susceptibility of patients with congenital neutropenia or chronic granulomatous disease [6,7,8]. Autosomal dominant hyper IgE syndrome (AD-HIES; also known as Job’s Syndrome), caused by dominant-negative, loss-of-function mutations in the Signal Transducer and Activator of Transcription 3 (STAT3) [9,10,11], is also characterized by *S. aureus* susceptibility, which exacerbates eczematoid dermatitis and causes pneumonia and recurrent skin abscesses in these patients. The effects of STAT3 dysfunction on epithelial antimicrobial function, including impaired differentiation of IL-17-secreting CD4+ T (Th17) cells [12,13], likely contribute to this susceptibility.

The skin microbiome of AD-HIES patients is altered compared to unaffected control subjects, with a significant increase in *Corynebacteria*, *Serratia*, and *Staphylococcus*, including *S. aureus*, *S. haemolyticus*, and *S. epidermidis* [14,15]. Molecular characterization of the staphylococcal strains present on the skin of AD-HIES patients has not been previously described. In particular, it remains unclear whether immunodeficiency selects for colonization by unique strains of *S. aureus*. Here, we identified the species of cultivable, mannitol-fermenting staphylococcal strains present on the skin of patients with AD-HIES. To determine whether patients with AD-HIES are colonized with particular *S. aureus* strains, all strains identified as *S. aureus* were genetically characterized by multi-locus sequence typing (MLST) and staphylococcal protein A (spa)-typing; assessed for the presence of *lukSF*-*PV*, *sek*, and *seq* virulence genes; tested for methicillin resistance; and assessed for their ability to lyse erythrocytes of human, sheep, and rabbit origin. We found a low diversity of sequence types, and an abundance of strains that expressed methicillin resistance, Panton-Valentine leukocidin (PVL), and staphylococcal enterotoxins K and Q (SEK, SEQ). Our results indicate that patients with AD-HIES may often carry antibiotic-resistant strains that harbor key virulence factors.

## 2. Results

### 2.1. Patients

We swabbed the antecubital fossa and volar forearm of 36 patients with AD-HIES and isolated mannitol-fermenting staphylococci for further characterization from 21 of these patients. The clinical characteristics of these 21 patients are presented in Table 1. These patients were followed at National Institutes of Health (NIH) and reside in 13 different states from western, mid-western, southern, and northeastern regions of the United States (US). There were 12 females (57%) and nine males (42%). Median age at time of swabbing was 23.8 years (range 1–53 years). All patients had STAT3 mutations confirmed by Sanger sequencing. The mutations were identified to be within the Src homology 2 (SH2) domain (*n* = 10; 48%), DNA-binding domain (*n* = 10; 48%), or transactivation domain (*n* = 1; 5%). Eighteen patients (86%) were taking oral anti-staphylococcal prophylactic antibiotics at the time of swabbing. These antibiotics included but were not limited to trimethoprim/sulfamethoxazole, clindamycin, amoxicillin-clavulanate, and cephalosporins.

Staphylococcal isolates from 13 patients with physician-diagnosed atopic dermatitis were included in the study. Patients with atopic dermatitis were chosen as a comparison group for the patients with AD-HIES since they were similarly seen at NIH, were also characterized by *S. aureus* skin colonization and infection, but did not have the dysfunctional STAT3-dependent immunodeficiency that defines AD-HIES. These patients represented residency in eight states from diverse regions of the US. Their clinical characteristics are presented in Table S1. The majority of patients were male (*n* = 10; 77%). The median age at time of swabbing was 16 years (range 3–51 years). Disease severity was assessed using the Scoring Atopic Dermatitis (SCORAD) index scale [16], and further classified as mild (SCORAD < 25), moderate (SCORAD 25–50), or severe (SCORAD > 50). Median SCORAD at time of swab was 16 (range 2–56). None of these patients had a history of skin abscesses or were on prophylactic antibiotics at the time of swabbing.

### 2.2. Strain Isolation and Identification

**Figure 1:**
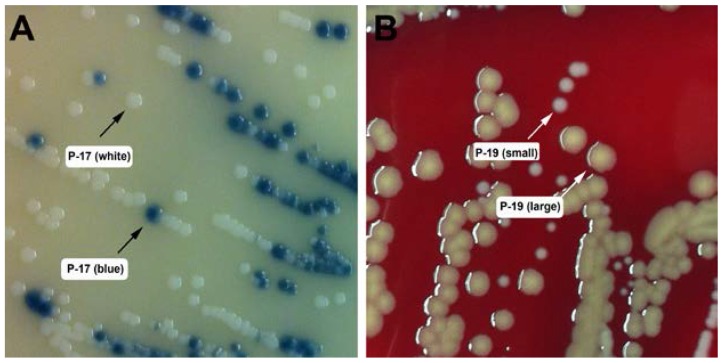
**Agar plate phenotypes of select staphylococcal isolates from patients with AD-HIES.** (**A**) Isolate P-17 on spectra MRSA plate. The blue colony represents a phosphatase-positive isolate that was confirmed to be *S. aureus*. The white colony represents a phosphatase-negative strain that was identified as *S. haemolyticus*. (**B**) Isolate P-19 on sheep blood agar plate. The large, yellow colony was identified as *S. aureus*; the small, white colony was identified as *S. hominis*.

To isolate *S. aureus* strains from individuals with AD-HIES, skin swabs were plated on mannitol salt agar (MSA). Mannitol-fermenting strains were isolated for further characterization. We obtained 25 mannitol-fermenting staphylococcal strains from the skin of 21 individuals with AD-HIES. An overview of all mannitol-fermenting strains isolated from these patients with AD-HIES is provided in Table 2 and Table 3. Two mannitol-fermenting staphylococcal strains were isolated from each of two individuals with AD-HIES, P-17 and P-19. These were designated P-17 (white) and P-17 (blue) for their appearance on Spectra MRSA plates (Figure 1A), or P-19 (large) and P-19 (small) for their colony size on blood agar (BA) plates (Figure 1B). Furthermore, two patients with AD-HIES (P-10 and P-25) were swabbed on two different occasions, and the resulting non-aureus staphylococcal (NAS) isolates were designated P-10a, P10b, P25a, and P25b, respectively (Table 3).

Of all 25 mannitol-fermenting strains isolated from patients with AD-HIES, 13/25 (52%) were *S. aureus*, as defined by coagulase-positivity (Table 2), and 12/25 (48%) were NAS, such as *S. haemolyticus* or *S. hominis*, determined by 16S rRNA sequencing (Table 3). We further determined that 7/13 (54%) *S. aureus* isolates were MRSA (Table 2). In comparison, 9/13 (59%) manntiol-fermenting isolates obtained similarly from patients with atopic dermatitis were *S. aureus*, and none of these were MRSA (Table S2).

### 2.3. Typing of S. aureus Isolates

MLST distinguishes bacterial strains based on DNA sequence variations in seven moderately conserved bacterial housekeeping genes (mlst.saureus.net). We identified only three different MLST types within all 13 *S. aureus* isolates from individuals with AD-HIES (Table 2). Of these, 61.5% (8/13) of strains were of Sequence Type (ST) 8. The second most frequent type was ST88 (3/13, 23%), accounting for three AD-HIES isolates. One strain (P-31) was non-typeable because of a unique MLST locus profile that did not match any entries in the mlst.net database (Table 2 and Table S3). To further assess the relatedness of strains, we performed spa typing [17] and identified six different spa types (Table 2). The most frequent spa type was t4013 (four isolates); these strains belonged to two MLST types, ST88 and ST2148 (Table 2 and Table S3). Similar analysis of the nine *S. aureus* isolates from our patients with atopic dermatitis revealed a more diverse distribution of MLST and spa types (Table S2).

### 2.4. Presence of the Phage-Encoded Toxins Panton-Valentine Leukocidin and Enterotoxins SEK, SEQ

**Figure 2:**
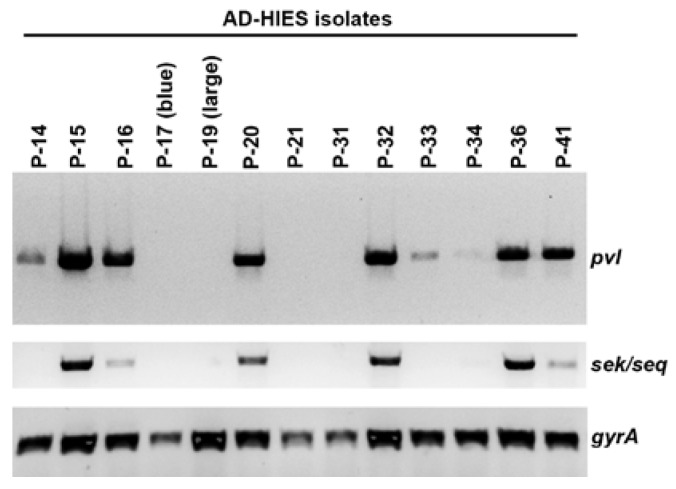
Detection of select toxin genes in *S. aureus* strains isolated from patients with AD-HIES. Presence of genes for Panton-Valentine leukocidin (*pvl*) and two superantigens (*sek*/*seq*) were assessed by PCR. As a DNA template control, the housekeeping gene encoding for gyrase A (*gyrA*) was partially amplified.

We tested all isolated *S. aureus* strains for the presence of the genes for *lukSF-PV*, *sek*, and *seq*, encoding for the two-component pore-forming cytotoxin Panton-Valentine leukocidin (PVL) [18] and for the staphylococcal enterotoxins K and Q (SEK, SEQ), respectively. These toxins are often found in USA300 strains that have been associated with CA-MRSA-associated outbreaks of skin and soft tissue infection. We found that a majority of *S. aureus* isolates from AD-HIES patients (9/13; 69%) contained the *lukF*/*lukS-PV* genes (Figure 2 and Table 2). Similarly, some AD-HIES patients harbored strains that were positive for the superantigen genes *seq*/*sek* (6/13; 46%). These toxin genes were found in only a striking minority of atopic dermatitis *S. aureus* isolates (Table S2).

### 2.5. Hemolysis on Sheep, Rabbit, and Human Agar Plates

**Figure 3:**
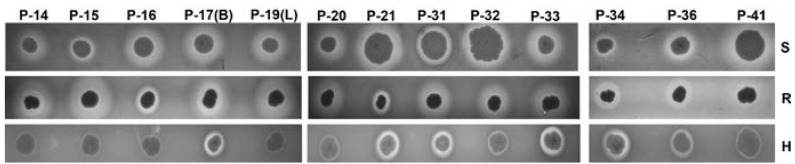
Hemolysis phenotype of *S. aureus* strains isolated from patients with AD-HIES. Hemolysis on sheep (S), rabbit (R), and human (H) blood agar is shown.

We phenotypically assessed the capacity of *S. aureus* isolates to lyse red blood cells, an activity ascribed to a number of staphylococcal virulence factors [19]. Because the ability of staphylococcal hemolysins to lyse erythrocytes exhibits species specificity [20], we characterized hemolysis on sheep, rabbit, and human BA plates. We found that all isolates lysed both sheep and rabbit erythrocytes (Figure 3). Robust lysis of human red blood cells was only evident for some strains (5/13; 38%), in particular those belonging to ST8, t064 type (P-17 (blue), P-21, P-34). Weak hemolysis of human BA plates was observed for all four t4013 isolates (P-14, P-15, P-16, P-19 (large)) as well as for the ST8, t008 isolates (P-20 and P-32).

## 3. Discussion

The primary aim of this study was to provide a detailed analysis of the *S. aureus* strains isolated from the skin of patients with AD-HIES. 16S rRNA sequencing has identified altered microbiomes in patients with AD-HIES [14,15], including the enrichment of *S. aureus* and non-aureus staphylococci. We sought to provide a more detailed, strain-specific analysis of AD-HIES-associated staphylococci, since *S. aureus* strains are known for their varied expression of colonization and virulence factors that can influence disease frequency and severity [2], and specific strains have been linked to skin infections [4].

We determined three different MLST sequence types in 13 *S. aureus* isolates from patients with AD-HIES. The most common sequence type was ST8, which accounted for eight of the 13 *S. aureus* isolates from patients with AD-HIES, and three of the nine *S. aureus* isolates from STAT3-sufficient patients with atopic dermatitis (3/9). These findings are consistent with ST8 being the most commonly isolated sequence type for MRSA in the US. [5]. The second most frequent sequence type isolated from our AD-HIES cohort was ST88, which has been reported in certain regions of Africa where it accounts for up to 83% of all MRSA isolates [21]. Of note, ST2148 was isolated from one patient with AD-HIES (P-15) and appears to be closely related to ST88, harboring the same spa-type and differing only at one MLST locus.

Among these same 13 AD-HIES-associated *S. aureus* isolates, we identified six unique spa-types, the most frequent ones being t4013 (4/13, 31%), t064 (3/13, 23%), and t008 (3/13, 23%). Spa-type t4013 was described in 2009 among MRSA strains isolated from wastewater in Sweden [22], and t064 and t008 are common types belonging to MLST ST8, the most frequently isolated ST of MRSA in the US from 2000 to 2013 [5]. Although conclusions are limited by our small sample size, taken together our data suggest that patients with AD-HIES are colonized with prevalent sequence types. We speculate the limited diversity may reflect the patients’ increased exposure to health care facilities and the selective pressure of prophylactic antibiotics.

*S. aureus* is known for its ability to acquire foreign DNA in the form of extra-chromosomal plasmids, chromosomally-integrated *S. aureus* pathogenicity islands (SaPI), and other phage-transported DNA regions. These DNA acquisition mechanisms give *S. aureus* a dynamic genetic variability that may confer advantages under certain circumstances, e.g., antibiotic resistance or acquisition of additional virulence factors [23]. In this study, we tested for the presence of four genes (*lukS-PV*, *lukF-PV*, *sek* and *seq*) and one resistance marker (methicillin) associated with the accessory gene regions SaPI1, PVL prophages, and staphylococcal cassette chromosome (SCC), respectively. Our findings suggest that patients with AD-HIES frequently carry methicillin-resistant, PVL-positive, and SEK/SEQ-positive strains, indicating significant virulence potential since PVL has been associated with worse outcomes during *S. aureus* pneumonia [24], and SEK and SEQ have been shown to contribute to T cell activation in a mouse model of pneumonia [25]. However, similar to observations in CA-MRSA infections, bacteremia and disseminated infections are a rare presentation of *S. aureus* in patients with AD-HIES, indicating that the immune dysfunction is largely limited to epithelial and mucosal sites.

*S. aureus* expresses a wide variety of pore-forming or membrane-damaging toxins that may influence virulence [20]. We evaluated the broad potential of the isolated strains to lyse blood from animal (sheep, rabbit) and human origin, and found that all strains similarly lysed rabbit and sheep blood. Differences were observed in the lysis of human blood. This might reflect the species-specific activity of alpha-hemolysin [20]. One might also speculate that the expression of PVL by some isolates inhibits the activity of other pore-forming toxins, such as LukED, as recently shown by Yoong and Torres [26], but a more detailed analysis of secreted amounts of PVL and LukED is needed to draw such conclusions and clarify the implications for clinical virulence.

To our knowledge, this is the first detailed characterization of *S. aureus* strains present on the skin of patients with AD-HIES. Our findings suggest that patients with AD-HIES are colonized with a variety of strains, including those that are known to be prevalent in the US. However, the diversity of AD-HIES strains appears limited compared to what might be expected from the wide number of circulating clinical strains [27,28] and our analysis of atopic dermatitis strains. A high percentage of AD-HIES isolates (7/13, 54%) displayed resistance to methicillin and encoded toxin genes that have been associated with both CA-MRSA strains and pneumonia. Our data suggest that the immunodeficiency of AD-HIES does not predispose to colonization with less virulent strains. In fact, the colonizing strains of patients seem to be enriched for virulence factors and antibiotic resistance, which is likely a reflection of exposure to health care settings and prophylactic antibiotics.

## 4. Materials and Methods

### 4.1. Ethics Statement

All subjects were evaluated at the NIH between 1 December 2013 and 30 June 2015. All subjects provided informed consent on clinical research protocols approved by the National Institute of Allergy and Infectious Diseases (NIAID) Institutional Review Board (clinical trials.gov identifiers: NCT00006150 and NCT02262819).

### 4.2. Collection and Isolation of Staphylococci

Subjects with AD-HIES (*n* = 36) or clinically diagnosed atopic dermatitis without a known genetic disorder (*n* = 13) were swabbed at the antecubital fossa and volar forearm, common sites of eczematous lesions colonized by *S. aureus* in AD-HIES as well as other forms of atopic dermatitis [14]. The swab was swirled in 2 mL of tryptic soy broth (TSB; Remel Thermo Scientific, Waltham, MA, USA). Aliquots were plated onto MSA (Remel Thermo Scientific) to select for *S. aureus*, which grows under high salt conditions and ferments mannitol. Unique mannitol-fermenting colonies, identified by their ability to produce yellow zones on MSA, were isolated, resuspended in TSB overnight, and plated onto sheep blood agar plates (Remel Thermo Scientific). *S. aureus* was identified by a positive slide coagulase test (Sigma-Aldrich, St. Louis, MO, USA). The species for coagulase-negative, mannitol-fermenting bacteria was identified by 16S rRNA sequencing. Subjects were designated by sequential numbering as they were enrolled. Their bacterial isolates were given corresponding numbers; AD-HIES isolates were preceded by ‘P’ (patient) and atopic dermatitis isolates were preceded by ‘AD,’ but overlapping numbers between groups were avoided to prevent confusion (Table 1).

### 4.3. DNA Isolation from Staphylococci and 16S rRNA Sequencing

DNA was isolated using a protocol described by Krausz and Bose [29]. The 16S rRNA gene was amplified with primers 16S-2F and 16S-1088R (Table 4) using Phusion DNA polymerase (Thermo Scientific) in a reaction volume of 50 μL. The amplification protocol was as follows: 98 °C for 40 s; 30 cycles of 98 °C for 10 s, 56 °C for 15 s, 72 °C for 20 s; and a final extension at 72 °C for 10 min. DNA was purified using the Qiagen PCR purification kit according to manufacturer’s recommendations (Qiagen, Valencia, CA, USA), and a minimum of two sequencing reactions per amplicon were performed (Eurofins MWG Operon, Huntsville, AL, USA) using both forward and reverse primers. Strain identification was performed using NCBI Blast.

### 4.4. Identification of MRSA Isolates

Mannitol-fermenting staphylococcal isolates were streaked onto Spectra MRSA (Remel Thermo Scientific) plates, which select for MRSA as navy blue colonies.

### 4.5. MLST and Spa Typing

Primer sequences for MLST typing were obtained from saureus.mlst.net and are listed in Table 4. Internal sequences of seven housekeeping genes were amplified using Phusion polymerase with the same protocol used for 16S rRNA sequencing. Purified PCR products were sequenced and sequences were trimmed according to saureus.mlst.net and compared to the MLST database using the single locus query option. Amplification and typing of the *spa* gene was performed with the same protocol used for 16S rRNA sequencing and primers spa-1113f and spa-1517r (Table 4). PCR products were sequenced (Eurofins MWG Operon) and spa types were identified using the spatyper (spatyper.fortinbras.us).

### 4.6. Screening for pvl, sek, and seq

The presence of the *lukSF-PV* and *sek*/*seq* genes was determined by PCR using primers listed in Table 4. The oligonucleotides for *sek*/*seq* were derived from the sequence of the USA300 clone FPR3757 [31] and anneal within the coding regions of both *sek* and *seq*. Both reactions were performed using Phusion polymerase with the following protocol: 98 °C for 40 s; 35 cycles of 98 °C for 10 s, 56 °C for 15 s, 72 °C for 30 s; and a final extension at 72 °C for 5 min.

### 4.7. Blood Agar Hemolysis

Bacteria were streaked from a glycerol stock onto sheep blood agar plates and incubated overnight at 37 °C. The next day, single colonies were spotted onto tryptic soy agar (TSA) plates containing 3% of sheep, rabbit (both from Quad Five, Ryegate, MT, USA), or human blood (Department of Transfusion Medicine, NIH, Bethesda, MD, USA). Plates were incubated overnight at 37 °C and photographed the next day.

## Figures and Tables

**Table 1:** Demographics and clinical characteristics of patients with AD-HIES.

Patient ID	Gender	Age ^+^	Race	STAT3 Mutation (Nucleotide; Amino Acid)	STAT3 Mutation Domain	History of:				On Antibiotics
						Eczema	Skin Abscess	PNA	CMC	
P-9	F	45	W	G1909A; V637M	SH2	Y	Y	Y	Y	N
P-10	M	27	B	C1144T; R382W	DNA	Y	Y	Y	Y	Y
P-14	F	45	W	T1151C; F384S	DNA	Y	Y	Y	Y	Y
P-15	F	18	W	C1144T; R382W	DNA	Y	Y	Y	Y	N
P-16	M	23	W	T1997G; L666R	SH2	Y	Y	Y	Y	Y
P-17	F	39	W	C2003T; S668F	SH2	Y	Y	Y	Y	Y
P-18	F	43	W	T1861G; F621V	SH2	Y	Y	Y	Y	Y
P-19	F	1	W	A1939G; N647D	SH2	Y	N	N	N	Y
P-20	M	7	B	C1144T; R382G	DNA	Y	Y	Y	Y	Y
P-21	F	13	W	T2117C; L706P	Trans-activation	Y	Y	Y	N	Y
P-25	M	44	W	1387delGTG; V463del	DNA	Y	Y	Y	Y	Y
P-26	M	9	B	A1831G; S611G	SH2	Y	Y	Y	N	Y
P-31	F	17	A	G1268A; R423Q	DNA	Y	N	N	N	Y
P-32	M	17	W	G1145A; R382Q	DNA	Y	Y	Y	Y	Y
P-33	M	6	A	G1909A; V637M	SH2	Y	Y	Y	N	Y
P-34	M	20	W	G1909A; V637M	SH2	Y	Y	Y	Y	Y
P-36	F	6	B	G1909A; V637M	SH2	Y	Y	Y	Y	Y
P-39	F	1	W	G1268A; R423Q	DNA	Y	N	N	N	Y
P-40	F	36	W	G1268A; R423Q	DNA	Y	Y	Y	Y	N
P-41	F	30	H	G1909A; V637M; G1381C; V461L	SH2	Y	Y	Y	Y	Y
P-53	M	53	W	G1145A; R382Q	DNA	Y	Y	Y	Y	Y

^+^ Age in years; M, male; F, female; A, Asian; B, Black; H, Hispanic; W, White; DNA, DNA binding domain; SH2, Src homology 2 domain; PNA, pneumonia; CMC, chronic mucocutaneous candidiasis; Y, yes; N, no.

**Table 2:** Coagulase-positive *S. aureus* strains isolated from patients with AD-HIES.

*S. aureus* Isolates from Patients with AD-HIES					
Strain ID	MLST ST	Spa Type	MRSA	PVL	SEK/SEQ
P-14	88	t4013	N	Y	N
P-15	2148	t4013	N	Y	Y
P-16	88	t4013	Y	Y	Y
P-17 (blue)	8	t064	Y	N	N
P-19 (large)	88	t4013	N	N	N
P-20	8	t008	Y	Y	Y
P-21	8	t064	N	N	N
P-31	N.D.	t189	N	N	N
P-32	8	t008	Y	Y	Y
P-33	8	t118	N	Y	N
P-34	8	t064	Y	Y	N
P-36	8	t451	Y	Y	Y
P-41	8	t008	Y	Y	Y

N.D., not determinable; ST, sequence type; N, no; Y, yes.

**Table 3:** Mannitol-fermenting, coagulase-negative staphylococcal strains isolated from patients with AD-HIES.

Non-Aureus Staphylococcal Isolates from Patients with AD-HIES	
P-9	*S. warneri*
P-10 a	*S. haemolyticus*
P-10 b	*S. hominis*
P-17 (white)	*S. haemolyticus*
P-18	*S. haemolyticus*
P-19 (small)	*S. hominis*
P-25 a	*S. haemolyticus*
P-25 b	*S. haemolyticus*
P-26	*S. simulans*
P-39	*S. hominis*
P-40	*S. hominis*
P-53	*S. haemolyticus*

**Table 4:** Oligonucleotide primers.

Name	5′–3′ Sequence	Purpose	Reference
16S-2F	GCRKGCYTAAYACATGCAAGTCGA	16S typing	This study
16S-1088R	CACGACACGAGCTGACGACAGCCA		
arcC-up	TTGATTCACCAGCGCGTATTGTC	MLST typing	saureus.mlst.net
arcC-dn	AGGTATCTGCTTCAATCAGCG		
aroE-up	ATCGGAAATCCTATTTCACATTC		
aroE-dn	GGTGTTGTATTAATAACGATATC		
glpf-up	CTAGGAACTGCAATCTTAATC		
glpf-dn	TGGTAAAATCGCATGTCCAATTC		
gmk-up	ATCGTTTTATCGGGACCATC		
gmk-dn	TCATTAACTACAACGTAATCGTA		
pta-up	GTTAAAATCGTATTACCTGAAGG		
pta-n	GACCCTTTTCTTCAAAAGCTTAA		
tpi-up	TCGTTCATTCTGAACGTCGTGAA		
tpi-dn	TTTGCACCTTCTAACAATTGTAC		
yqiL-up	CAGATACAGGACACCTATTGGC		
yqiL-dn	CGTTGAGGAATCGATACTGGAAC		
spa-1113f	TAAAGACGATCCTTCGGTGAGC	*spa* typing	www.ridom.de
spa-1514r	CAGCAGTAGTGCCGTTTGCTT		
PVL-1	ATGTCTGGACATGATCCA	*LukSF-PV* amplification	[30]
PVL-2	AACTATCTCTGCCATATGGT		
SEQ/SEK1	GTATGGCGGAATTACGTTGG	*sek*/*seq* amplification	This study
SEQ/SEK2	TTGGTAACCCATCATCTCCTG		

## Supplementary Materials

The following are available online at http://www.mdpi.com/2076-0817/6/2/23/s1. Table S1. Demographics and clinical characteristics of patients with physician-diagnosed atopic dermatitis. Table S2. Coagulase-positive *S. aureus* strains isolated from patients with atopic dermatitis. Table S3. Single locus typing results for all *S. aureus* isolates.
