# Supplementary material for: Molecular Typing of Staphylococcus aureus Isolated from Patients with Autosomal Dominant Hyper IgE Syndrome
Source: Pathogens. 2017 Jun 6;6(2):23. doi: 10.3390/pathogens6020023 (PMC5488657; doi:10.3390/pathogens6020023)
Supplement: Supplementary file 1 [file pathogens-06-00023-s001.pdf]

Table S1. Demographics and clinical characteristics of patients with physician-diagnosed atopic dermatitis

Patient ID	Gender	Age+	Race	SCORAD at time of culture	AD severity	History of skin abscesses	On prophylactic antibiotics
AD-1	M	11	B	18	mild	N	N
AD-2	M	46	W	9	mild	N	N
AD-3	M	21	A	16	mild	N	N
AD-4	F	19	W	16	mild	N	N
AD-5	F	12	B	8	mild	N	N
AD-6	M	6	W	14	mild	N	N
AD-7	F	16	W	2	mild	N	N
AD-13	M	14	W	ND	mild	N	N
AD-22	M	51	W	13	mild	N	N
AD-29	M	5	A	45	moderate	N	N
AD-30	M	3	A	56	severe	N	N
AD-35	M	16	A	33	moderate	N	N
AD-37	M	19	W	56	severe	N	N

+Age in years; M, male; F, female; A, Asian; B, Black; W, White; SCORAD, Scoring Atopic Dermatitis index scale; AD, atopic dermatitis; ND, not determined; N, no

Table S2. Coagulase-positive *S. aureus* strains isolated from patients with atopic dermatitis

***S. aureus* isolates from patients with atopic dermatitis**					
**ID**	**MLST ST**	**Spa type**	**MRSA**	**PVL**	**SEK/SEQ**
AD-1	59	t12548	N	N	Y
AD-2	8	t1476	N	N	N
AD-3	N.D.	t267	N	Y	N
AD-4	72	t148	N	N	N
AD-7	1191	t2260	N	N	N
AD-13	72	t148	N	N	N
AD-29	8	t118	N	N	N
AD-30	8	t118	N	Y	N
AD-37	N.D.	t922	N	N	N

N.D., not determinable; ST, sequence type; N, no; Y, yes

Table S3. Single locus typing results for all *S. aureus* isolates

ID	*arcC*	*aroE*	*glpF*	*gmk*	*pta*	*tpi*	*yqiL*	ST
***S. aureus* isolates from patients with atopic dermatitis**								
AD-1	19	23	15	2	19	20	15	59
AD-2	3	3	1	1	4	4	3	8
AD-3	3	(1)*	1	1	1	5	3	N.D.
AD-4	1	4	1	8	4	4	3	72
AD-7	3	3	1	1	4	5	3	1991
AD-13	1	4	1	8	4	4	3	72
AD-29	3	3	1	1	4	4	3	8
AD-30	3	3	1	1	4	4	3	8
AD-37	1	1	1	119	1	1	1	N.D.
***S. aureus* isolates from patients with AD-HIES**								
P-14	22	1	14	23	12	4	31	88
P-15	3	1	14	23	12	4	31	2148
P-16	22	1	14	23	12	4	31	88
P-17 (blue)	3	3	1	1	4	4	3	8
P-19 (large)	22	1	14	23	12	4	31	88
P-20	3	3	1	1	4	4	3	8
P-21	3	3	1	1	4	4	3	8
P-31	3	1	1	8	1	4	1	N.D.
P-32	3	3	1	1	4	1	3	8
P-33	3	3	1	1	4	4	3	8
P-34	3	3	1	1	4	4	3	8
P-36	3	3	1	1	4	4	3	8
P-41	3	3	1	1	4	4	3	8

*This *aroE* gene has a 99.38% similarity to allele 1, but contained the single nucleotide polymorphism T305G. ST, sequence type. N.D., not determinable
